# Dual-backbone pectic polysaccharide activates NOD1/RIPK2/NF-κB and mitochondrial metabolism to enhance the immune activity of macrophages

**DOI:** 10.1038/s41538-025-00570-0

**Published:** 2025-10-08

**Authors:** Ziwei Liu, Hangyu Li, Shuyao Yang, Xinnan Zhang, Qianqian Liu, Yuan Xu, Yanwen Yang, Yi Liao, Yao Wang, Haibo Feng

**Affiliations:** 1 College of Animal Husbandry and Veterinary Medicine, Southwest Minzu University, Chengdu, Sichuan PR China; 2 Institute of Qinghai-Tibetan Plateau, Southwest Minzu University, Chengdu, Sichuan PR China

**Keywords:** Carbohydrates, Drug discovery, Immunology, NMR spectroscopy, Biochemistry, Glycobiology

## Abstract

A novel pectic polysaccharide with dual backbones (SHP-A, 15,866 Da) was isolated from Sinopodophyllum hexandrum fruits. This polysaccharide is a heteropolymer composed of six monosaccharides. Structural characterization based on nuclear magnetic resonance spectroscopy, methylation analysis, and other methods demonstrated that SHP-A features a unique architecture consisting of a rhamnogalacturonan-I backbone (alternating α-GalpA and α-Rha molecules with β-Galp/α-Araf side chains) and a β-glucomannan backbone (alternating β-Manp and β-Glcp molecules), interconnected via β-Galp bridges. Bioactivity assays showed that SHP-A exerts potent immunomodulatory effects by enhancing macrophage phagocytosis and nitric oxide release. Transcriptomics analyses and NOD1 inhibitor interventions confirmed that SHP-A can activate the NOD1/RIPK2/NF-κB axis and induce M1 polarization in RAW264.7 macrophages, thereby upregulating various cytokines and chemokines. Furthermore, SHP-A also appeared to enhance immune function through mitochondrial metabolism, which indicated its potential as an immunomodulatory agent that can be used in the development of immune-enhancing health foods in the future.

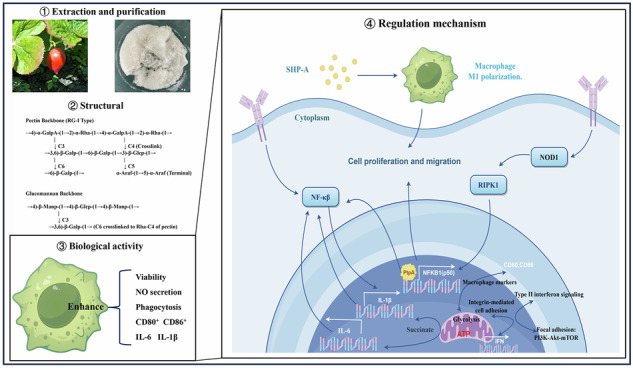

## Introduction

Phytopolysaccharides, which are macromolecular compounds composed of monosaccharide units linked via glycosidic bonds, can demonstrate either homogeneous or heterogeneous structural arrangements. These naturally occurring polymers exhibit broad-spectrum biofunctionalities, including immunomodulation, reactive oxygen species scavenging, pathogen inhibition, glucose homeostasis regulation, viral suppression, and anti-tumor effects. Thus, they have emerged as a research hotspot in not only food science but also animal husbandry and aquaculture. Accumulating evidence shows that polysaccharides have several special biological activities^1^, which are closely related to their complex spatial structure. Therefore, research on the structure of polysaccharides has received renewed attention. However, progress in this area is hindered by persistent technical bottlenecks and scientific challenges. Specifically, the highly branched, heterogeneous architectures and dynamic conformational shifts of polysaccharides make it exceptionally difficult to precisely elucidate their monosaccharide composition, linkage patterns, and three-dimensional arrangements. Current analytical techniques, such as nuclear magnetic resonance (NMR), mass spectrometry (MS), and atomic force microscopy (AFM), often require multi-platform integration. However, they show insufficient sensitivity for trace samples and poor resolution for insoluble polysaccharides. Moreover, microheterogeneity-induced inter-batch variations further complicate the process of structural characterization based on these techniques. Meanwhile, the absence of systematic theoretical frameworks that link 3D topological features with bioactivities, coupled with the lack of unified structural classification standards and shared databases, severely restricts mechanistic interpretation and translational application. Despite these technical challenges in structural analyses, the intrinsic correlations between the “structural codes” of polysaccharides and their biological functions continue to drive technological innovations in analytical techniques and foster interdisciplinary collaboration, which is of profound significance for diverse fields, including food science, aquaculture, and biomedical research.

*Sinopodophyllum hexandrum* is a plant primarily distributed in Gansu, Qinghai, Shaanxi, Yunnan, Sichuan, and Tibet. The ovoid fruit of this plant is bright red when ripe and is traditionally picked and consumed directly by local communities as a wild fruit^2^. In recent years, these local communities have recognized the value of this fruit and cultivated it as an economic crop^3^. Interestingly, evidence shows that a key polysaccharide derived from *S. hexandrum* fruit is a polymeric carbohydrate composed of multiple monosaccharides. This polysaccharide possesses a host of biological activities, including blood sugar regulation, immune system modulation, as well as antiviral and antioxidant effects, indicating broad prospects for further research^4^.

Here, we extracted and purified the polysaccharide from *S. hexandrum* fruits and structurally characterized it. Further, we examined its effects on proliferation, phagocytosis, and nitric oxide (NO) release in mouse macrophages. In addition, we also used transcriptome sequencing to investigate the mechanism through which this polysaccharide regulates immune activity in RAW264.7 mouse macrophages. Our findings could guide further studies on this polysaccharide and provide ideas for research on its immunomodulatory effects.

## Results

### Polysaccharide purification

**Fig. 1:**
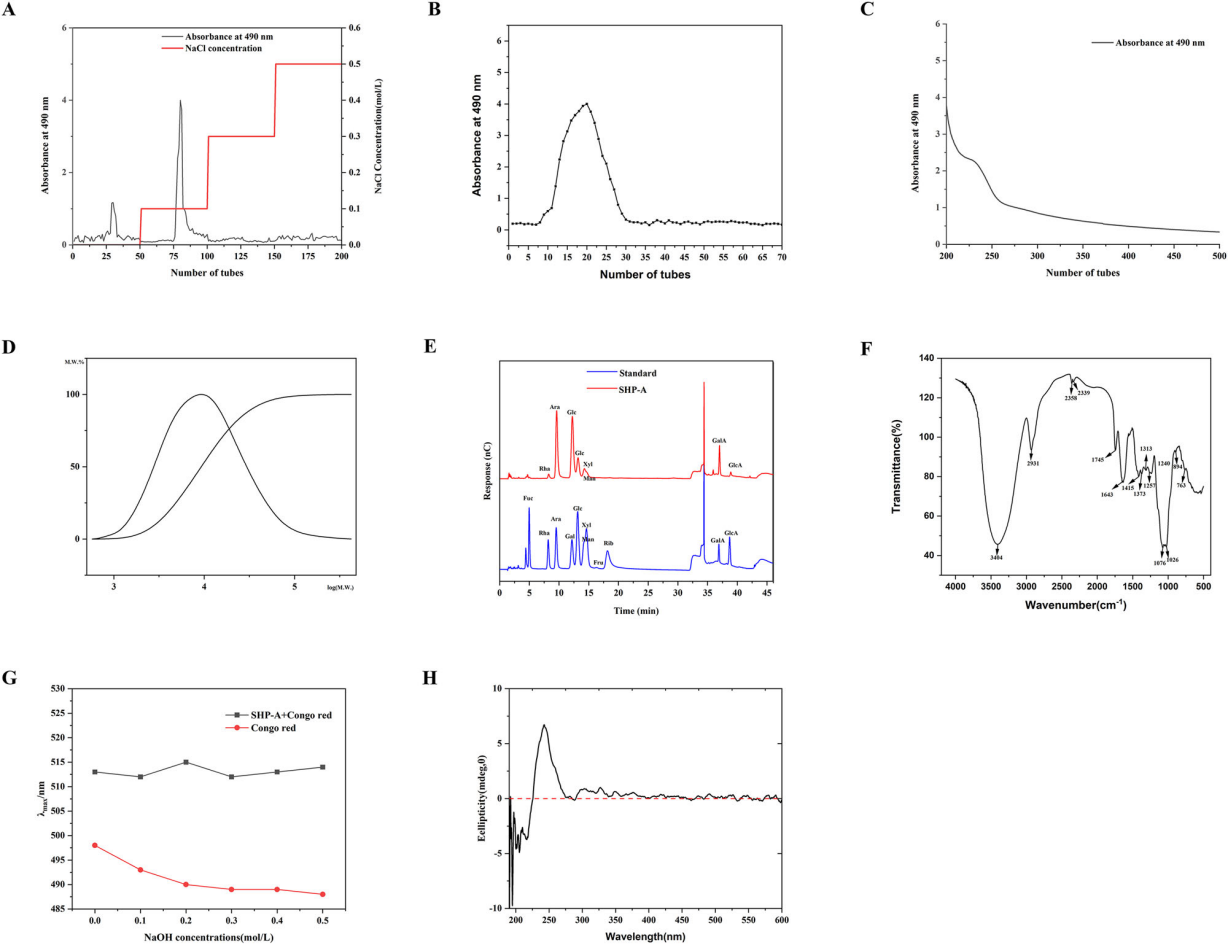
Chemical properties and characteristics of SHP-A. **A** Cellulose elution curve of SHP-A. **B** Gel elution curve of SHP-A. **C** Molecular weight distribution curve of SHP-A. **D** UV spectra of SHP-A. **E** Ionic chromatogram of SHP-A. **F** FT-IR spectra of SHP-A. **G** Wavelength of maximum absorption of SHP-A in the presence of Congo red under different NaOH concentrations. **H** CD spectra of SHP-A.

As shown in Fig. 1A, B, the deproteinized polysaccharides were initially purified using a DEAE-52 cellulose column. The highest polysaccharide content was observed in tubes 51–100, eluted with 0.1 M NaCl. The extract from tube 51–100 was concentrated, freeze-dried, and re-purified using a CL-6B gel column. The polysaccharide solution from tubes 5–30 was collected, concentrated, and freeze-dried to obtain the refined polysaccharide (SHP-A). SHP-A showed no absorbance at a wavelength of 280 nm, which indicated that this polysaccharide fraction did not contain any protein (Fig. 1C).

### Molecular weight and composition

SHP-A exhibited a homogeneous molecular weight distribution, with a Mw of 15.866 Da in Table 1 (Fig. 1D). Ion chromatography (Fig. 1E) revealed the presence of eight monosaccharides, mainly arabinose, galactose, glucose, and galacturonic acid. The molar ratios of these monosaccharides are summarized in Table 2.

**Table 1:** GPC calculation results

Analysis item	Numerical value
Number average molecular weight(Mn)	6349
Weight average molecular weight(Mw)	15,866
Z average molecular weight(Mz)	46,358
Z + 1 average molecular weight(Mz1)	116,780
Mw/Mn	2.49902
Mz/Mw	2.92186

**Table 2:** Analysis of monosaccharides

Name	Sample peak area	Sample retention time	Reference material peak area	Reference material retention time	Sample molar ratio
Fuc	0	0	13.434	4.967	0
Rha	2.63	8.192	9.645	8.175	0.0226
Ara	52.98	9.542	16.11	9.525	0.2986
Gal	57.103	12.175	14.379	12.167	0.3005
Glc	19.944	13.142	29.794	13.133	0.0507
Man	9.11	14.200	11.07	14.308	0.0623
Xly	4.101	14.500	25.116	14.6	0.0148
Fru	0	0	0.899	16.308	0
Rib	0	0	18.988	18.133	0
GalUA	10.97	36.942	3.299	36.9	0.2335
GluUA	1.935	38.850	7.987	38.717	0.0170

### FT-IR analysis

The FT-IR spectrum of SHP-1 contained characteristic absorption bands corresponding to key functional groups (Fig. 1F). The broad and intense absorption band at 3404 cm^−^^1^ was attributed to the O–H stretching vibrations of hydroxyl groups (-OH)—a hallmark of saccharide ring structures. The peak observed at 2931 cm^−^^1^ corresponded to C–H stretching vibrations, which primarily originated from the -CH and -CH₂ groups in the glycosyl units^5^. Meanwhile, the absorption bands at 1643 cm^−^^1^ and 1415 cm^−^^1^ were assigned to the asymmetric (νₐs) and symmetric (νs) stretching vibrations of carboxylate groups (COO^−^), respectively. This suggested the presence of uronic acid residues such as glucuronic and galacturonic acid, indicating that SHP-1 was an acidic polysaccharide^6^. Finally, a distinct peak near 894 cm^−1^, characteristic of β-glycosidic linkages, confirmed the existence of the β-configuration in the glycosidic bonds of SHP-A^7^.

### Congo red test

Congo red forms a complex with polysaccharides containing a triple helix structure, which leads to a redshift in their *λ*_max_. As shown in Fig. 1G, the *λ*_max_ of SHP-A did not change significantly in the presence of 0–0.5 mol/L NaOH. This indicated that SHP-A has a random helical conformation rather than a triple helical structure. CD spectra analysis was performed to further confirm these findings.

### CD spectra analysis

Tertiary structure is a key determinant of the biological activity of polysaccharides. Upon hydration, polysaccharide chains undergo conformational transitions, which are driven by intrachain interactions and manifest as structural irregularities (e.g., helical coiling and chain entanglement). These anisotropic molecular arrangements induce chiroptical asymmetry, ultimately generating characteristic Cotton effects in aqueous solutions^8^. As shown in Fig. 1H, in the range of 213–282 nm, the CD spectrum of SHP-A presented a typical cotton effect. This showed that SHP-A has a relatively stable tertiary structure^9^, likely spiral in nature.

### Methylation analysis

Methylation analysis is an important component of polysaccharide structural characterization. Coupled with GC-MS, this method reveals the relative abundance and linkage positions of the sugar residues in polysaccharides. Following the reduction, methylation, hydrolysis, and acetylation of the polysaccharide sample, GC-MS analysis was performed in this study. Through comparison with standard spectral libraries, eleven monosaccharide residues were identified: Araf-(1→, →5)-Araf-(1→, Galp-(1→, →3)-Galp-(1→, →4)-Manp-(1→, →4)-Galp-(1→, →4)-Glcp-(1→, →2)-Galp-(1→, →6)-Galp-(1→, →3,4)-Galp-(1→ and→3,6)-Galp-(1→. Tables 3 and 4 demonstrate alterations in monosaccharide composition before and after polysaccharide reduction, while Table 5 provides detailed information on their relative proportions and linkage configurations.

**Table 3:** Analysis of monosaccharides before reduction

Name	Sample peak area	Sample retention time	Reference material peak area	Reference material retention time	Sample molar ratio
Fuc	0	0	13.434	4.967	0
Rha	2.63	8.192	9.645	8.175	0.0226
Ara	52.98	9.542	16.11	9.525	0.2986
Gal	57.103	12.175	14.379	12.167	0.3005
Glc	19.944	13.142	29.794	13.133	0.0507
Man	9.11	14.200	11.07	14.308	0.0623
Xly	4.101	14.500	25.116	14.6	0.0148
Fru	0	0	0.899	16.308	0
Rib	0	0	18.988	18.133	0
GalUA	10.97	36.942	3.299	36.9	0.2335
GluUA	1.935	38.850	7.987	38.717	0.0170

**Table 4:** Analysis of monosaccharides after reduction

Name	Sample peak area	Sample retention time	Reference material peak area	Reference material retention time	Sample molar ratio
Fuc	0	0	13.434	4.967	0
Rha	1.523	8.208	9.645	8.175	0.016
Ara	46.631	9.533	16.11	9.525	0.328
Gal	63.865	12.192	14.379	12.167	0.440
Glc	18.047	13.142	29.794	13.133	0.059
Man	6.429	14.267	11.07	14.308	0.058
Xly	3.526	14.525	25.116	14.6	0.017
Fru	0	0	0.899	16.308	0
Rib	0	0	18.988	18.133	0
GalUA	2.44	36.625	3.299	36.9	0.082
GluUA	0	0	7.987	38.717	0

**Table 5:** Analysis of partially methylated alditol acetates in polysaccharide structural characterization

Methylated sugar	Mass fragments (m/z)	Molar ratio	Type of linkage
2,3,5-Me3-Araf	43, 71, 87, 101, 117, 129, 145, 161	0.170	Araf-(1→
2,3-Me2-Araf	43, 71, 87, 99, 101, 117, 129, 161, 189	0.368	→5)-Araf-(1→
2,3,4,6-Me4-Galp	43, 71, 87, 101, 117, 129, 145, 161, 205	0.041	Galp-(1→
2,4,6-Me3-Galp	43, 87, 99, 101, 117, 129, 161, 173, 233	0.013	→3)-Galp-(1→
2,3,6-Me3-Manp	43, 87, 99, 101, 113, 117, 129, 131, 161, 173, 233	0.041	→4)-Manp-(1→
2,3,6-Me3-Galp	43, 87, 99, 101, 113, 117, 129, 131, 161, 173, 233	0.113	→4)-Galp-(1→
2,3,6-Me3-Glcp	43, 87, 99, 101, 113, 117, 129, 131, 161, 173, 233	0.066	→4)-Glcp-(1→
3,4,6-Me3-Galp	43, 87, 101, 129, 161, 189	0.060	→2)-Galp-(1→
2,3,4-Me3-Galp	43, 87, 99, 101, 117, 129, 161, 189, 233	0.026	→6)-Galp-(1→
2,6-Me2-Galp	43, 87, 99, 117, 129, 149	0.017	→3,4)-Galp-(1→
2,4-Me2-Galp	43, 87, 117, 129, 159, 189, 233	0.084	→3,6)-Galp-(1→

A subset of the detected Galp-(1 → 4) and Galp-(1 → 3,4) linkages originated from reduced galacturonic acid residues, reflecting modifications during the derivatization process.

### 1D and 2D NMR spectroscopy

**Fig. 2:**
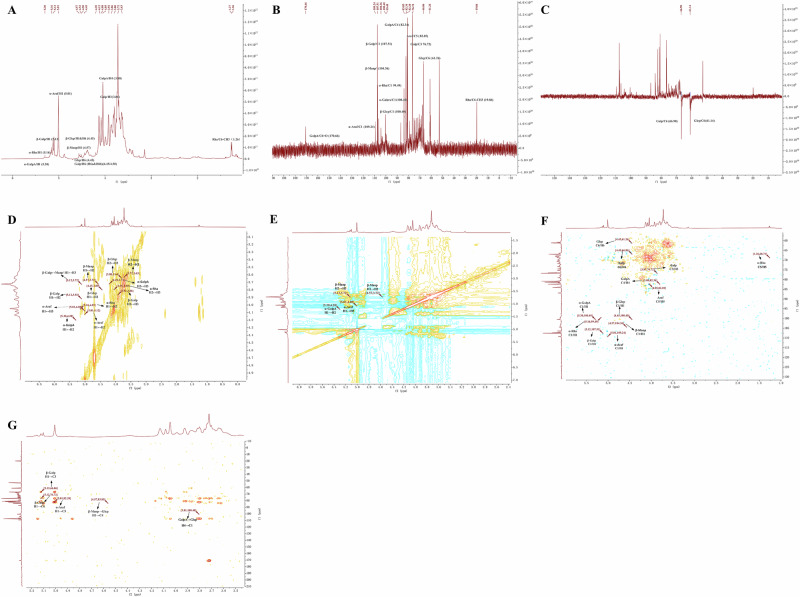
NMR spectra of SHP-A. **A**^1^H NMR spectra of SHP-A. **B**^13^C NMR spectra of SHP-A. **C** DEPT-based analysis of SHP-A. **D** COSY spectra of SHP-A. **E** NOESY spectra of SHP-A. **F** HSQC of SHP-A. **G** HMBC of A SHP-A.

As shown in Fig. 2A–G, the anomeric signals of α-galacturonic acid (α-GalpA) (C1/H1: δ 100.40/5.30) and the glycosidic linkage at C4 (C4/H4: δ 82.34/3.80) unequivocally demonstrated →4)-α-GalpA-(1 → 2) connectivity in SHP-A. This was further supported by the long-range correlation between H1 (δ 5.30) and C4 (δ 82.34) in the HMBC spectrum^10^. Adjacent α-rhamnose (α-Rha) exhibited anomeric resonance at C1/H1 (δ 99.49/5.16), and the C2 glycosidic bond (δ 75.19) was confirmed via HSQC (C1/H1: 99.49/5.16) and HMBC (H1 → C2: δ 75.19), establishing the →2)-α-Rha-(1 → 4)-α-GalpA-(1→ backbone sequence. Notably, substitution by the →3,6)-β-Galp side chain at Rha-C3 was validated based on chemical shift perturbations at C3/H3 (δ 76.72/3.80) and the HMBC correlation between β-Galp H1 (δ 5.12) and Rha-C3 (δ 76.72)^4^. The crosslinking between Rha-C4 (δ 80.9) and β-Galp-C6 (δ 66.86) in the glucomannan side chain was corroborated by NOE interactions (H4 [δ 3.80] ↔ H6 [δ 4.45/4.50]) in NOESY and HMBC-derived coupling (β-Galp-C6 [δ 66.86] ↔ Rha-C4 [δ80.9])^11^. For the branched galactan (→3,6)-β-Galp-(1 → 6)-β-Galp-(1 → 3)-β-Glcp-(1→), the anomeric signal (C1/H1: δ 107.51/5.12) and substitution patterns at C3 (δ 76.72) and C6 (δ 66.86) were assigned via HSQC (C1/H1: 107.51/5.12) and HMBC (H1 → C3/C6: δ 76.72/66.86), while the linkage between β-Galp-C6 (δ 66.86) and the downstream β-Galp-C1 residue (δ 104.36) was evidenced based on the identified correlations (H6 [δ 4.45] ↔ C1 [δ 104.36])^12^. The α-configuration of the terminal arabinan (α-Araf-(1 → 5)-α-Araf) was confirmed by its anomeric signals (C1/H1: δ 109.24/5.01) and C5 linkage (C5/H5: δ 82.20/4.05), with COSY sequential correlations (H1 [δ 5.01] → H2 [δ 4.12] → H3 [δ 3.98] → H4 [δ 3.75] → H5 [δ 4.05]) confirming the furanose conformation. In the glucomannan backbone, alternating →4)-β-Manp-(1 → 4)-β-Glcp-(1→ units were identified based on anomeric resonance (β-Manp: C1/H1 δ 104.36/4.57; β-Glcp: C1/H1 δ 100.40/4.45), HSQC assignments, and HMBC correlations (H1 → C4: δ 83.85)^13^. The substitution at Glcp-C6 (δ 61.26) was validated by HSQC (H6 [δ 4.45] ↔ C6 [δ 61.26]). Finally, crosslinking via 3,6)-β-Galp was confirmed by HMBC (H1 [δ 5.12] → C3 [δ 74.20]) and the spatial proximity between β-Galp-H6 (δ 4.45/4.50) and Rha-H4 (δ 3.80) in the NOESY spectrum^14^. The NMR chemical shift assignments of SHP-A are shown in Table 6

**Table 6:** NMR chemical shift assignments of SHP-A

Unit	Site	δ13 C	δ1H	DEPT	Link	HMBC	HSQC	NOESY	COSY
α-GalpA	C1/H1	100.40	5.30	CH	→4)-GalpA	H1 → C4 (82.34)	C1 ↔ H1 (100.40/5.30)	H1 ↔ H2 (4.20)	H1 ↔ H2 (4.20)
	C4/H4	82.34	3.80	CH	-	-	C4 ↔ H4 (82.34/3.80)	-	H4 ↔ H5 (4.08)
α-Rha	C1/H1	99.49	5.16	CH	→2)-Rha	H1 → C2 (75.19)	C1 ↔ H1 (99.49/5.16)	H1 ↔ H2 (4.05)	H1 ↔ H2 (4.05)
	C2/H2	75.19	4.05	CH	-	-	C2 ↔ H2 (75.19/4.05)	H2 ↔ H3 (3.80)	H2 ↔ H3 (3.80)
β-Manp	C1/H1	104.36	4.57	CH	→4)-Manp	H1 → C4 (83.85)	C1 ↔ H1 (104.36/4.57)	H1 ↔ H3 (3.72)	H1 ↔ H2 (3.72)
	C4/H4	83.85	3.80	CH	-	-	C4 ↔ H4 (83.85/3.80)	H4 ↔ H5 (4.08)	H4 ↔ H5 (4.08)
β-Glcp	C1/H1	100.40	4.45	CH	→4)-Glcp	H1 → C4 (83.85)	C1 ↔ H1 (100.40/4.45)	-	H1 ↔ H2 (3.80)
	C6/H6	61.26	4.45	CH2	→6)-Glcp	-	C6 ↔ H6 (61.26/4.45)	H6a ↔ H6b (4.50)	H6a ↔ H6b (4.50)
β-Galp	C1/H1	107.51	5.12	CH	→3,6)-Galp	H1 → C3 (76.72), C6 (66.78)	C1 ↔ H1 (107.51/5.12)	H1 ↔ H3 (3.86)	H1 ↔ H2 (3.92)
	C6/H6	66.86	4.45/4.50	CH2	→6)-Galp	-	C6 ↔ H6 (66.86/4.45)	H6a ↔ H6b	H6a ↔ H6b
α-Araf	C1/H1	109.24	5.01	CH	→5)-Araf	H1 → C5 (82.20)	C1 ↔ H1 (109.24/5.01)	H1 ↔ H5 (4.08)	H1 ↔ H2 (4.12)
	C5/H5	82.20	4.05	CH	-	-	C5 ↔ H5 (82.20/4.05)	H5 ↔ H1 (5.01)	H5 ↔ H1 (5.01)

### Appearance and structure

**Fig. 3:**
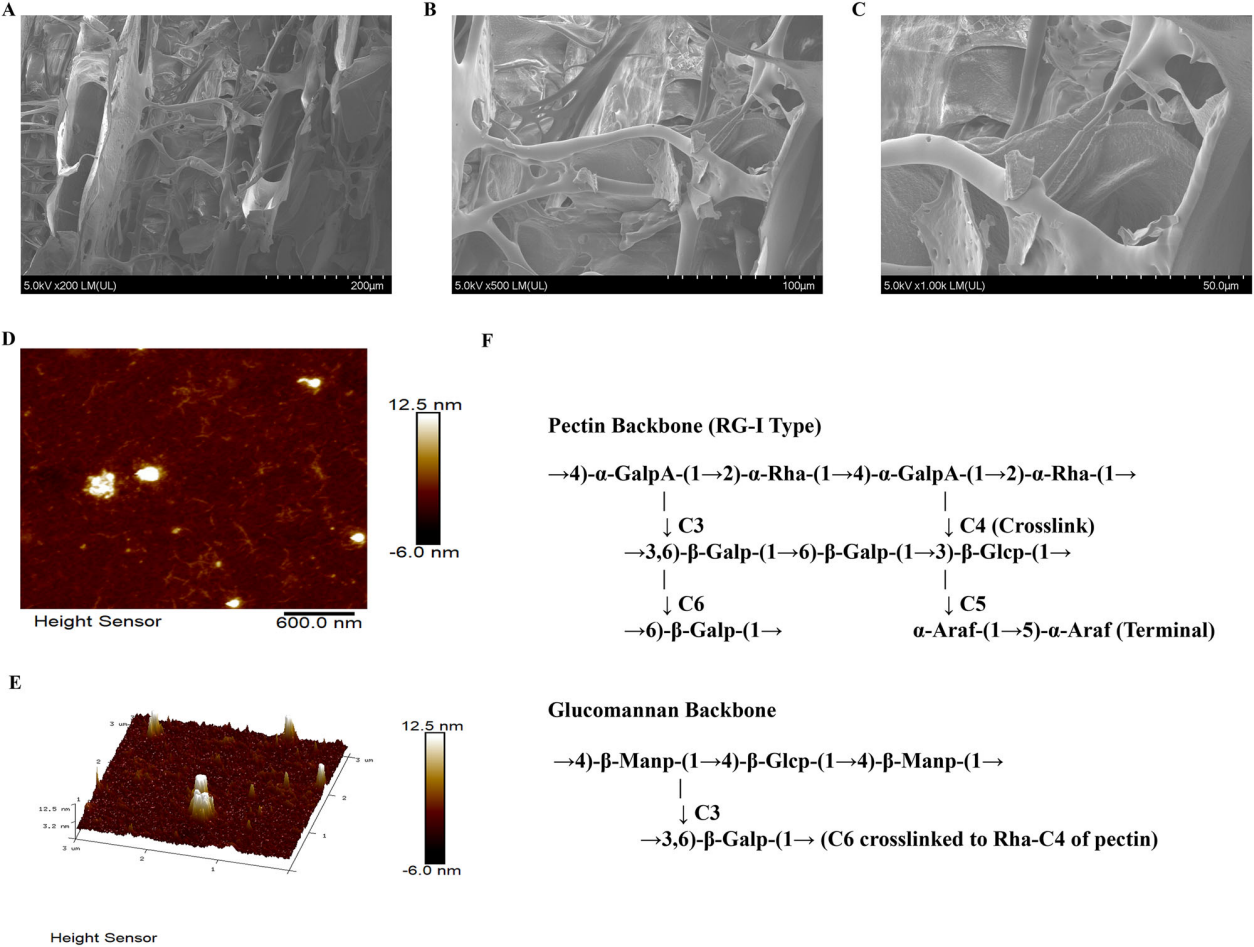
Scanning electron micrographs and atomic force micrographs of SHP-A. **A**–**C** Scanning electron micrographs of SHP-A at different magnifications. **D**, **E** 3D and planar AFM images of SHP-A. **F** Proposed structure of SHP-A.

Based on the results of the aforementioned experiments, we speculated that the polysaccharide architecture of SHP-A consists of two distinct backbones^13^: (1) a pectin backbone (RG-I type) with alternating →4)-α-GalpA-(1 → 2)-α-Rha-(1→ units, bearing a branched galactan side chain (→3,6)-β-Galp-(1 → 6)-β-Galp-(1 → 3)-β-Glcp-(1 →) at Rha-C3 and a crosslinking β-Galp-(1→ chain at Rha-C4; and (2) a glucomannan backbone composed of →4)-β-Manp-(1 → 4)-β-Glcp-(1→ repeats, which is connected to the pectin backbone via the 3,6)-β-Galp side chain (C6 → Rha-C4). Moreover, the results indicated the presence of terminal α-Araf-(1 → 5)-α-Araf units appended to the galactan branch. The proposed structure is shown in the Fig. 3F. SEM showed that SHP-A exhibits an irregular lamellar morphology (Fig. 3A–C), with regions of varying thickness. Pores of diverse dimensions were also observed across both the surface and cross-sectional views of SHP-A. Three-dimensional and planar AFM images (Fig. 3D, E) revealed a fibrous network architecture, with occasional spherical and non-spherical protrusions measuring 3.2–12.5 nm in height. Overall, SHP-A demonstrated an average surface roughness of 0.891 nm. The interconnected polysaccharide branches formed molecular aggregates through entanglement, enhancing the structural stability of the polysaccharide^15^.

### Viability, NO release, and phagocytosis activity in mouse macrophages

**Fig. 4:**
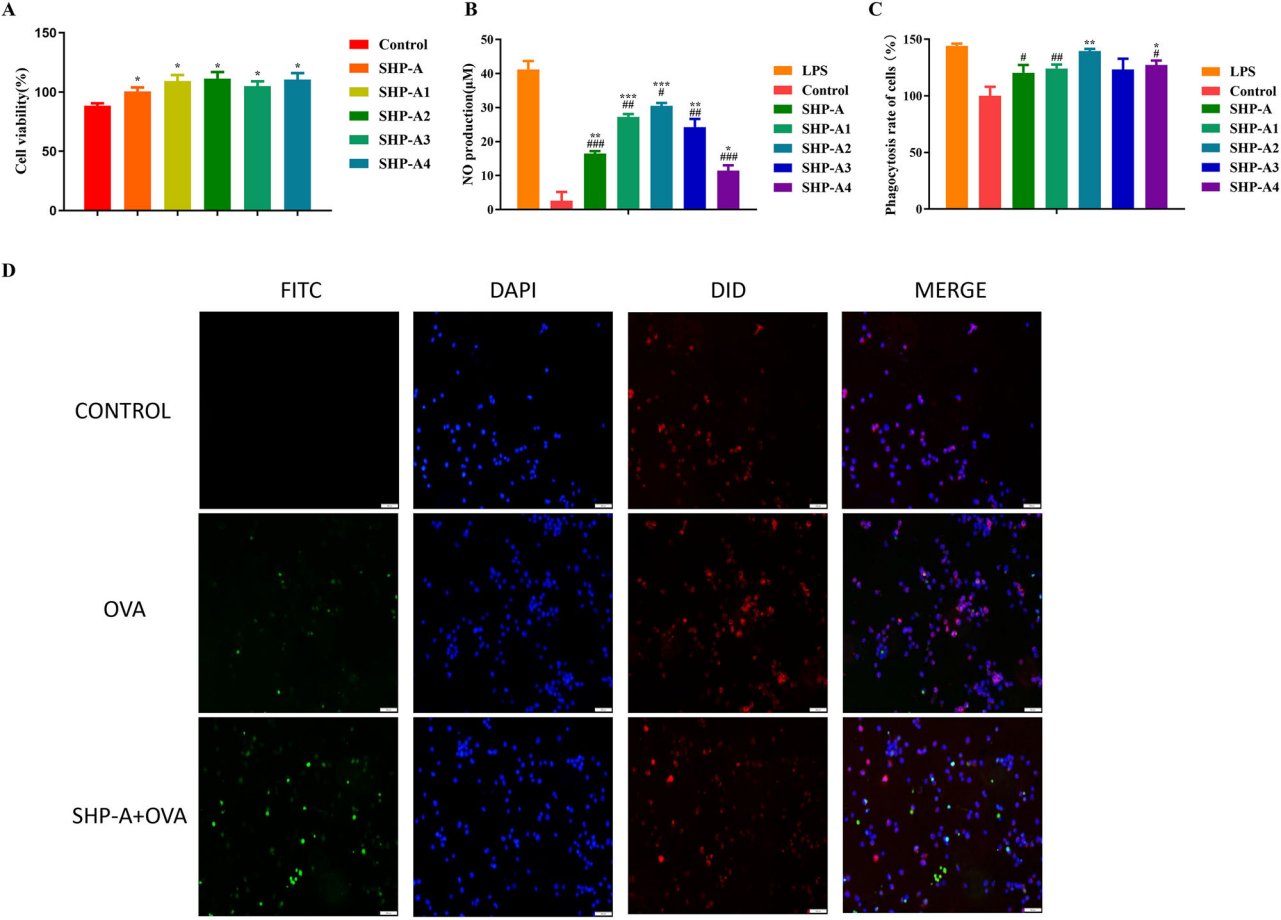
Effect of SHP-A on cell proliferation, NO release, phagocytic activity, and OVA phagocytosis rates in mouse macrophages. **A** Proliferation rate. **B** NO production. **C** Phagocytic activity. **D** Fluorescence images showing the effect of SHP-A on OVA phagocytosis in mouse macrophages. Green fluorescence represents FITC-labeled OVA, while blue fluorescence represents DAPI-labeled nuclei and red fluorescence represents DID-labeled cell membranes. The overlap of all three signals yields orange signals.

Figure 4A demonstrates that SHP-A, within a concentration range of 250–1000 μg/mL, exerted no cytotoxic effects against mouse macrophages. However, it significantly enhanced cellular proliferation in these cells (*P > 0.05 versus the untreated control group). Activated macrophages exert antimicrobial activity through NO release. As shown in Fig. 4B, all SHP-A-treated groups displayed markedly higher NO release than the control group (*P < 0.05). Notably, the difference between the SHP-A-treated and control groups became increasingly more statistically significant as the SHP-A concentration rose from 250 μg/mL to 1000 μg/mL, but was limited at lower SHP-A treatment concentrations. Conversely, the comparison between SHP-A-treated and LPS-stimulated groups revealed an inverse trend, with the smallest significant difference observed at 250 μg/mL (#*P* < 0.05), coinciding with the highest NO production. These results suggested that SHP-A effectively potentiates NO production in murine macrophages, with 250 μg/mL SHP-A serving as the optimal concentration in this context. Additionally, the neutral red phagocytic capacity of macrophages was also enhanced significantly after treatment with 250 μg/mL SHP-A (Fig. 4C), showing highly significant differences compared to the untreated control (*P < 0.05) but no statistical variance relative to the LPS group (#*P* < 0.05). To corroborate phagocytic enhancement at this treatment concentration, FITC-labeled OVA was employed for fluorescence microscopy analysis. As depicted in Fig. 4D, SHP-A treatment markedly increased green fluorescence signals in mouse macrophages. Multichannel overlay images further revealed a greater intensity of orange puncta in the FITC-OVA-SHP-A group versus the FITC-OVA group, indicating enhanced OVA internalization, predominantly localized to the perinuclear regions. Collectively, these data highlighted the immunomodulatory efficacy of SHP-A in augmenting phagocytotic activity and inflammatory mediator secretion in macrophages^16^.

### GO and macrophage polarization analysis

**Fig. 5:**
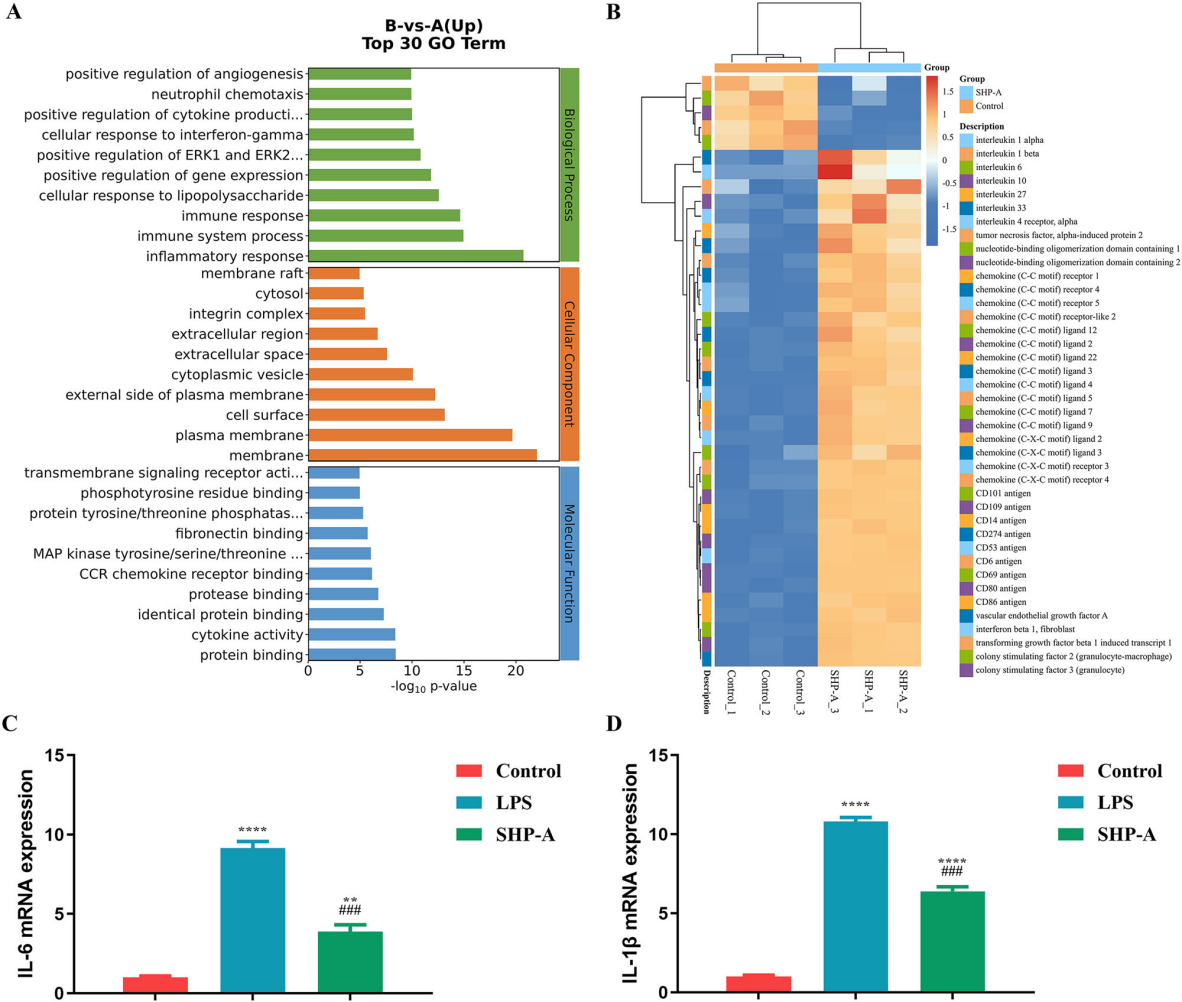
GO and immune-related genes clustering heatmap analysis of macrophages following SHP-A treatment. **A** GO diagram and **B** clustering heatmap for analyzing differentially expressed genes in RAW264.7 mouse macrophages treated with SHP-A (250 μg/mL) for 12 h. **C** mRNA expression of IL-6. **D** mRNA expression of IL-1β.

GO is a comprehensive database that describes gene functions under three key modules: biological processes, cell components, and molecular functions. In this study, the GO enrichment analysis of differentially expressed genes (DEGs) revealed several up-regulated GO terms in each of the three modules (Fig. 5A). Of these, most terms were directly related to immunity, especially in the biological processes and molecular functions modules^17^.

Macrophages can carry out multiple functions by changing their phenotypes in response to changes in their micro-environment through a process called macrophage polarization. M1 macrophages are characterized by the secretion of a large number of inflammatory factors, such as TNF-α, IL-1β, IL-23, IL-6, GM-CSF (CSF2), and IL-12^18^. M1 macrophages can also secrete co-stimulatory factors such as CD80^+^ and CD86^+^ and show strong antigen presentation ability. In contrast, M2 macrophages are characterized by the expression of IL-10 and the chemokines CCL1, CCL17, CCL18, CCL22, and CCL24, and demonstrate anti-inflammatory, angiogenic, and tissue repair functions^19^. The gene enrichment results in Fig. 5B show that the up-regulated genes related to M1 polarization in treated mouse macrophages were those governing the production of the co-stimulatory factors CD80 and CD86; the pro-inflammatory factors IL-1β, IL-1α, and IL-6; the chemokine CCL5; and the granulocyte macrophage colony-stimulating factors CSF2 and NOS2^20^. Meanwhile, fewer DEGs related to M2 polarization were detected. However, the pro-inflammatory factors IL-10 and IL-4Rα and chemokine VEGF-A were up-regulated, while TGFb1I1 was down-regulated. Collectively, these findings showed that SHP-A can induce M1 polarization in RAW264.7 mouse macrophages.

**Fig. 6:**
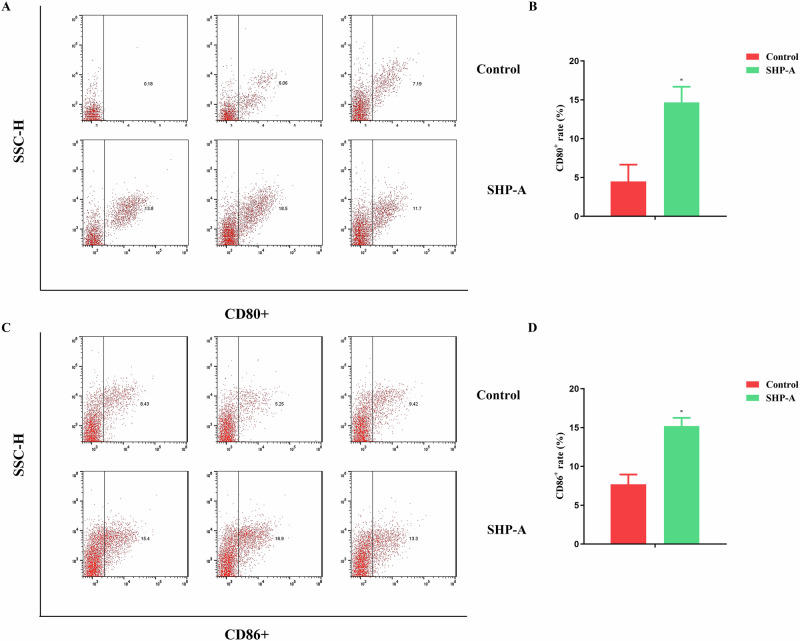
Expression of CD80^+^ and CD86^+^ on surface of RAW264 7 mouse macrophages. **A**, **B** Expression of CD80^+^. **C**, **D** Expression of CD86^+^.

Moreover, enrichment for genes related to NO was also detected after SHP-A treatment; specifically, NOS1 was down-regulated and NOS2 was up-regulated. Thus, SHP-A appeared to increase NO release in macrophages by promoting the expression of the NOS2 gene. Following drug treatment, the mRNA expression of IL-6 and IL-1β in macrophages was quantified using RT-qPCR (Fig. 5C, D), while the surface expression of M1-associated co-stimulatory molecules CD80 and CD86 was assessed through flow cytometry (Fig. 6A–D)^21^. The results demonstrated that SHP-A significantly upregulated IL-6 and IL-1β mRNA levels (*P* < 0.05) as well as CD80^+^ and CD86^+^ surface marker expression in macrophages. These findings were consistent with the results of transcriptomic analysis, collectively demonstrating that SHP-A potentiates macrophage polarization toward the M1 phenotype and enhances immunological activation^22^.

### KEGG enrichment analysis and immunoregulatory effects of SHP-A on macrophages

**Fig. 7:**
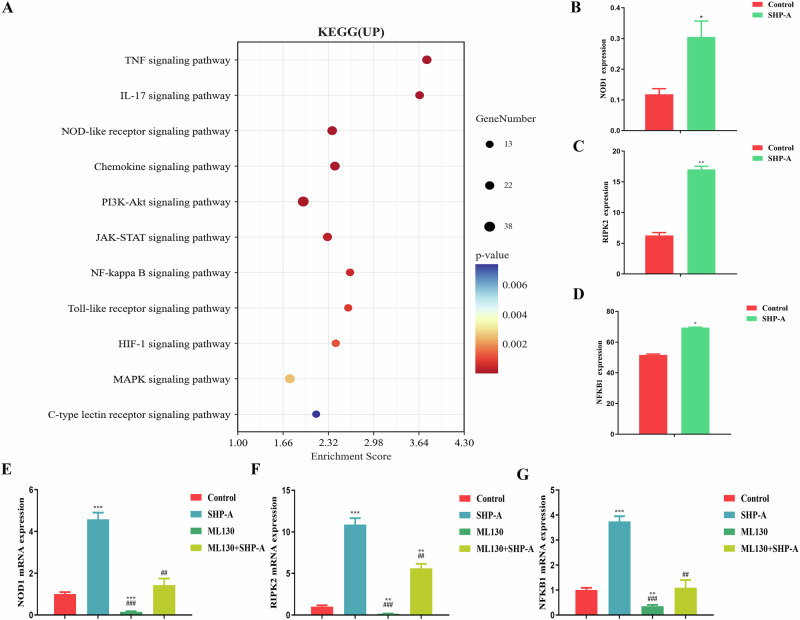
Analysis and mechanistic validation of immune-related KEGG pathways in macrophages following SHP-A treatment. **A** KEGG enrichment analysis of up-regulated genes. **B** Expression of NOD1. **C** Expression of RIPK2. **D** Expression of NFKB1. **E** mRNA expression of NOD1 after SHP-A treatment. **F** mRNA expression of RIPK2 after SHP-A treatment. **G** mRNA expression of NFKB1 after SHP-A treatment.

The NF-κB signal pathway is the hub of cytokine storms^18^. NF-κB is a pivotal transcription factor that directly mediates the expression of multiple pro-inflammatory factors, including cytokines (e.g., TNF-α, IL-6, IL-1β), chemokines, and inflammatory mediators (e.g., COX-2, iNOS). The excessive release of these molecules by immune cells triggers dysregulated inflammatory cascades, culminating in a cytokine storm. Furthermore, NF-κB serves as a convergent downstream effector for a range of immune signaling pathways (e.g., TLR, NLR, TNFR, and IL-1R), integrating pathogen recognition, cellular damage signals, and pro-inflammatory stimuli. Its activation involves the Iκκ complex, which phosphorylates and degrades the inhibitory protein IκB, enabling the nuclear translocation of NF-κB and subsequent transcriptional initiation^23^. This leads to a self-amplifying feedback loop (e.g., TNF-α/IL-1β-induced NF-κB reactivation), which exacerbates inflammatory signaling. Meanwhile, the concomitant suppression of anti-inflammatory factors (e.g., IL-10) disrupts immune homeostasis, ultimately driving pathological tissue damage. The KEGG pathway analysis of DEGs (Fig. 7A) revealed enrichment for the Toll-like receptor signaling pathway and NOD-like receptor signaling pathway, which can transmit information to activate the NF-κB signaling pathway. Analysis of transcriptomics data showed that although no obvious change in the expression of the core Toll-like receptor signaling gene TLR4 expression was detected, NOD1 and NOD2—involved in the NOD-like receptor signaling pathway—were significantly up-regulated^24^. Further data analysis showed that SHP-A can significantly increase the expression of NOD1, RIPK2, and NFKB1 (Fig. 7B–D). NOD1, a member of the nucleotide-binding and oligomerization domain (NOD)-like receptor (NLR) family, is established as a critical mediator in host defense. It participates in immune responses against diverse pathogens. In mammals, studies have demonstrated that upon activation, the previously inactive NOD1 receptor undergoes a conformational change. This transition facilitates ATP-dependent oligomerization of its NACHT domain. The signal is subsequently relayed to the caspase activation and recruitment domain (CARD) located at the N-terminus. This CARD domain then engages in homotypic interaction with the CARD domain of the receptor-interacting serine-threonine kinase 2 (RICK2/RIPK2) adapter protein. This cascade ultimately culminates in the activation of the NF-κB signaling pathway, triggering transcriptional activation of target genes, and the maturation of proinflammatory cytokines such as interleukin-1β (IL-1β)^25–27^. Therefore, we set up a blank group, inhibitor group (ML130), and SHP-A treatment group. The mRNA expression levels of NOD1, RIPK2, and NFKB1 in different groups of cells were quantified through RT-qPCR. Following suppression of NOD1 mRNA expression in macrophages by the inhibitor, mRNA levels of RIPK2 and NFKB1 were also significantly reduced. Notably, treatment with SHP-A resulted in markedly higher mRNA expression of NOD1, NFKB1, and RIPK2 compared to the inhibitor group, with no significant difference observed relative to the control group (Fig. 7E–G). These findings robustly demonstrate the modulatory effect of SHP-A on NOD1, indicating that SHP-A primarily regulates the immune response via the NOD1/RIPK2/NF-κB axis.

### Effects of SHP-A on mitochondrial metabolism

**Fig. 8:**
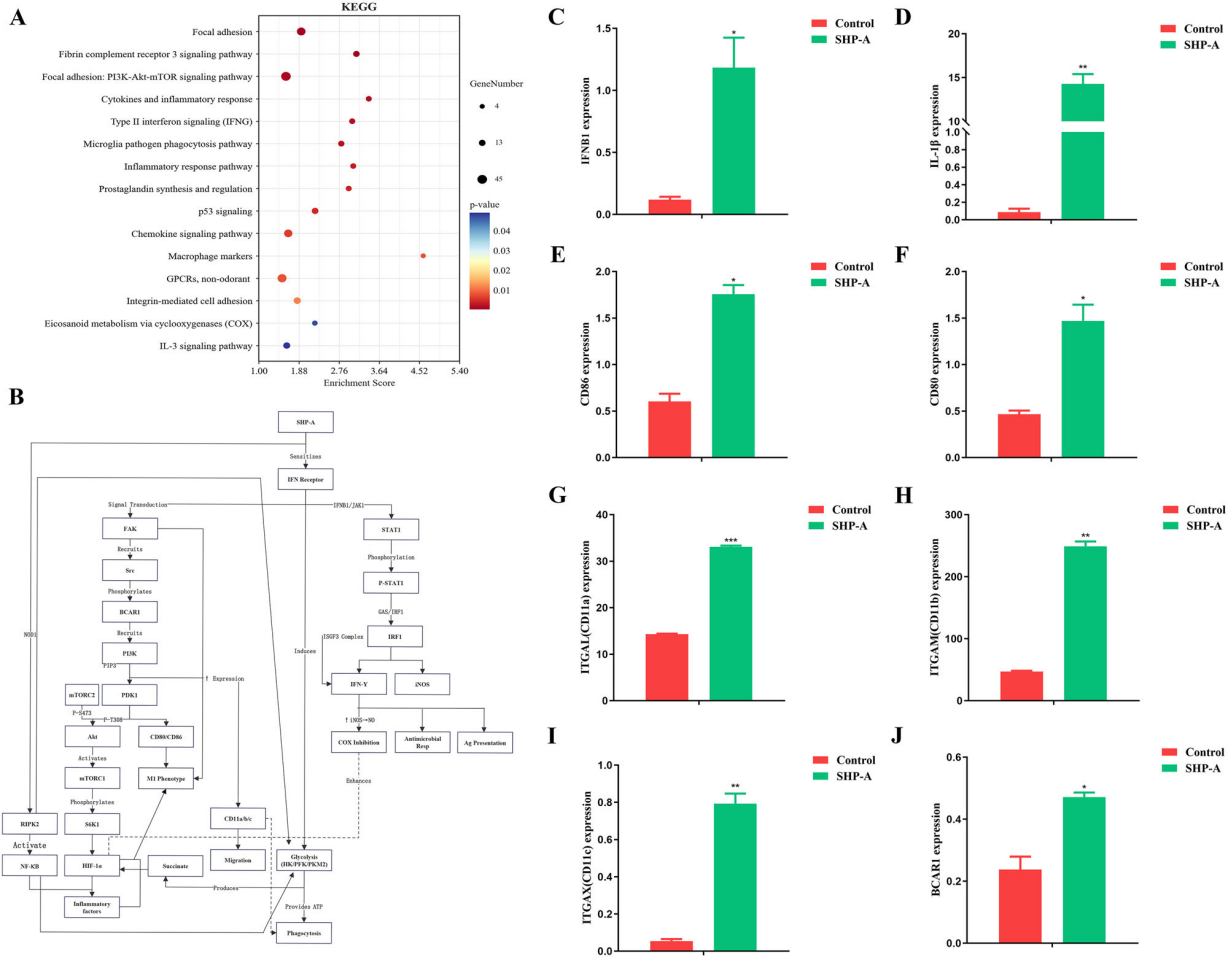
Analysis of immune-associated mitochondrial metabolic pathways in response to SHP-A treatment. **A** KEGG enrichment analysis of metabolism-related genes. **B** Metabolic network diagram. **C** Expression of IFNB1. **D** Expression of IL-1β. **E** Expression of CD86. **F** Expression of CD80. **G** Expression of ITGAL. **H** Expression of ITGAM. **I** Expression of ITGAX. **J** Expression of BCAR1.

Mitochondrial metabolism is a critical contributor to immunoregulation in macrophages. Our WikiPathways bubble plot analysis (Fig. 8A) led to the identification of pathways such as Focal adhesion, Focal adhesion: PI3K-Akt-mTOR signaling pathway, Type II interferon signaling (IFNG), Macrophage markers, and Integrin-mediated cell adhesion, which have been linked directly to mitochondrial metabolism-dependent immune modulation in macrophages^28^. Glycolytic reprogramming is a metabolic hallmark of M1 macrophages. Upon LPS/IFN-γ stimulation, glycolysis leads to rapid ATP production, and glycolytic intermediates (e.g., succinate) enhance the expression of pro-inflammatory cytokines such as IL-1β. The activation of the PI3K-Akt-mTOR pathway amplifies glycolytic flux, which is associated with M1 phenotypic markers (CD86, CD80) and integrin-dependent macrophage migration and phagocytic activity. Notably, focal adhesion kinase facilitates M1 polarization by stimulating the PI3K-Akt-mTOR cascade to promote glycolysis. Furthermore, IFN-γ augments glycolysis by suppressing mitochondrial respiratory chain complex IV (e.g., cytochrome c oxidase) while simultaneously enhancing antimicrobial responses and antigen presentation. The data from this study revealed that SHP-A could significantly up-regulate key components, including IFNB1 (within type II interferon signaling), macrophage markers (CD86/CD80), immunoregulatory integrins (ITGAX [CD11c], ITGAM [CD11b], ITGAL [CD11a]), and the focal adhesion adapter protein BCAR1 (p130Cas) (Fig. 8C–J), in macrophages^29^. Beyond these findings, established literature confirms that RIPK2—when engaged by NOD1—forms a critical nexus linking innate immunity to glycolytic reprogramming. This receptor-interacting kinase not only potentiates glycolysis through NF-κB pathway activation, but also orchestrates transcriptional upregulation of glycolytic enzymes. The consequent acceleration of glycolytic flux generates substantial ATP, enhancing macrophage phagocytic potency while driving robust succinate accumulation. Crucially, succinate synergizes with PI3K-Akt-mTOR signaling to stabilize HIF-1α, which transactivates pro-inflammatory mediators, thereby promoting M1-like polarization and substantially augmenting immunometabolic activation^30–33^. Collectively, these findings demonstrated that SHP-A selectively drives M1 polarization by orchestrating mitochondrial metabolism–immune system crosstalk, thereby exerting immunomodulatory effects. The metabolic network diagram is provided in Fig. 8B.

## Discussion

In this study, a novel dual-backbone pectic polysaccharide (SHP-A) was isolated from the fruits of *S. hexandrum* and structurally characterized as a heteropolymeric complex composed of a rhamnogalacturonan-I backbone bearing galactan/arabinofuranosyl side chains and a β-glucomannan backbone interconnected via β-galactopyranosyl bridging motifs. Advanced spectroscopic and microscopic techniques, including FT-IR, NMR, and AFM, revealed the irregular porous morphology and stable non-triple-helical conformation of SHP-A, as well as its distinct β-glycosidic linkages and carboxylate group signatures, confirming its acidic nature. Functionally, SHP-A exhibited robust immunomodulatory activity by significantly enhancing phagocytic activity and NO production in macrophages, while up-regulating key M1 polarization cytokines (IL-1β, IL-6) and surface co-stimulatory markers (CD80⁺, CD86⁺). Transcriptomic profiling and pathway enrichment analysis highlighted that the NOD1/RIPK2/NF-κB signaling axis was the central driver of macrophage M1 polarization following SHP-A treatment. The pharmacological inhibition of NOD1 significantly attenuated NFKB1 mRNA expression, mechanistically validating the identified regulatory cascade. In addition, transcriptomic analysis indicated that SHP-A can also regulate macrophage M1 polarization and enhance immune function through mitochondrial metabolism. Our findings revealed that the structural intricacy of SHP-A, particularly its dual-backbone architecture and β-galactan crosslinking, likely underpins its enhanced bioactivity, highlighting its potential as a promising functional food additive or immunotherapeutic agent. This study not only provides methodological guidance for resolving the topologies of complex dual-backbone polysaccharides but also offers a theoretical and methodological foundation for developing natural immunomodulators and functional foods.

## Methods

### Materials

*S. hexandrum* fruits were purchased from Cangxitang Biotechnology Co., Ltd. (Chengdu, China). Chemical reagents (Na₂SO₄, phenol, NaCl, H₂SO₄, HCl, NaOH, trichloroacetic acid, and KH₂PO₄) and chromatographic materials (DEAE cellulose-S28556 and D101 macroporous resin) were obtained from Kelong Chemical Co., Ltd. (Chengdu, China) and Yuanye Biotechnology Co., Ltd. (Shanghai, China), respectively. Monosaccharide standards, FITC, and DAPI were provided by Yuanye Biotechnology Co., Ltd. Some specialized reagents were also procured, such as PMP, CHCl₃, NaN₃, and CH₃CN (Aladdin Biochemical Technology Co., Ltd., Shanghai, China); TFA (Sinopharm Chemical Reagent Co., Ltd., Shanghai, China); Congo red (Macklin Biochemical Co., Ltd., Shanghai, China); and DID (C-reagent Biotechnology Co., Ltd., Shanghai, China). Cell culture components (DMEM, CCK-8, PBS) were sourced from Boster Biological Technology Co., Ltd. (Wuhan, China), while the NO assay kit was purchased from Yuhengsheng Material Technology Co., Ltd. (Suzhou, China). Finally, LPS, DMSO and ML130 (NOD1 inhibitor) were obtained from Solarbio Technology Co., Ltd. (Beijing, China), and reverse transcription reagents were sourced from Dalian Bao Biological Co., Ltd. (Dalian, China).

### Polysaccharide purification

The crude polysaccharide obtained from *S. hexandrum* fruits via ethanol precipitation was subjected to sequential deproteinization and decolorization. Further purification was achieved through DEAE-52 cellulose and Sepharose CL-6B column chromatography. The polysaccharide was eluted with distilled water and a stepwise gradient of NaCl solutions (0.1, 0.3, and 0.5 M) at a rate of 1 mL/min, with the volume of each fraction being 10 mL. Fractions demonstrating homogeneous molecular weight distribution were pooled and lyophilized to obtain the *S. hexandrum* polysaccharide (SHP-A).

### Protein detection

Polysaccharide aqueous solution sample were subjected to full-wavelength scanning (200–300 nm) using a UV spectrophotometer (UV-5200, METASH, China).

### Fourier transform infrared spectroscopy (FT-IR)

Potassium bromide (KBr) and polysaccharide powder were ground evenly and pressed into thin slices using a tablet press. Infrared scanning analysis was performed in the range of 400–4000 cm^−1^ (Cary660, China).

### NMR

Each sample (40 mg) was dissolved in D_2_O and analyzed using an AVⅡ-600 MHz NMR spectrometer (Bruker, Switzerland).

### Molecular weight analysis

The molecular weight of the polysaccharide was determined using GPC, with 0.05 M Na_2_SO_4_ and 0.02% NaN_3_ serving as the mobile phase. Chromatographic conditions included a column temperature of 40 °C and an injection volume of 500 μL (Agilent, USA).

### Monosaccharide analysis

A 500 mM NaOH/50 mM NaOAc aqueous solution (20.5 g NaOAc and 1.3 mL 50% NaOH in 500 mL) and a 20 mM NaOH solution (1.04 mL 50% NaOH diluted to 1 L (water) were prepared and stored at room temperature(25 °C). Monosaccharide standards (5 mg each) and samples (5 mg) were hydrolyzed with 2 mL of 3 M TFA at 120 °C for 3 h, dried under nitrogen, and reconstituted in 5 mL of water. The standards were diluted to prepare mixed calibration solutions, while the samples were diluted 10-fold and centrifuged (12,000 rpm for 5 min) for analysis. Separation was performed on a Dionex CarbopacTM PA10 column (4 × 250 mm) using mobile phases A (H_2_O), B (500 mM NaOH/50 mM NaOAc), and C (20 mM NaOH) under gradient elution conditions (0–30 min: 100% C → 50% A/50% C; 30.1–46 min: 70% A/30% B). The flow rate was 1.0 mL/min, column temperature was 30 °C, and injection volume was 25 μL; furthermore, electrochemical detection was employed. The calculation method for monosaccharide molar ratio is calculated according to the following Eqs. (1) and (2):

1 $${\rm{Sample\; Monosaccharide\; Content}}=({\rm{Sample\; Peak\; Area}}/{\rm{Standard\; Peak\; Area}})\times {\rm{Standard\; Concentration}}$$

2 $${\rm{Molar\; Amount\; Calculation}}={\rm{Sample\; Monosaccharide\; Content}}/{\rm{Molar\; Mass}}$$

The molar ratio of monosaccharides in the sample is derived from the ratio of their calculated molar amounts.

### Methylation analysis via GC–MS

Polysaccharide samples (15–20 mg) were methylated with reagents A/B in DMSO under ultrasonication. The reaction was carried out at 30 °C for 60 min with continuous magnetic stirring and terminated through the addition of ultrapure water. The mixture was dialyzed, and the product was lyophilized. Methylation efficiency was confirmed with FT-IR using KBr pellets. Methylated polysaccharides were hydrolyzed with 2 M TFA (90 min), reduced using NaBH_4_, acetylated with acetic anhydride, and purified via CH_2_Cl_2_ extraction. GC-MS (HP-INNOWAX column, 30 m × 0.32 mm × 0.25 μm) was performed using He as the carrier gas (1 mL/min). The temperature was increased from 140 to 230 °C at a rate of 1 °C/min, and the temperature of the injector/detector was maintained at 250 °C. The polysaccharide sample (80 mg) was reduced using a Automatic reduction of carboxyl groups instrument (Borui Sugar Biotechnology, BR-HYY-001, China). The sample was weighed into a beaker, dissolved in distilled water, and then treated with a uronic acid activator. The pH of the reductant instrument was adjusted to 4.6 and the reaction was allowed to proceed for 3 h. Subsequently, the pH was adjusted to 6.8 and the reaction continued for an additional 2 h. The sample was then concentrated and dialyzed using a 1000 Da molecular weight cut-off (MWCO) dialysis membrane. This entire procedure (dissolution, activation, reduction at pH 4.6 and 6.8, concentration, and dialysis) was repeated 3–5 times. The final product was lyophilized. The resulting sample was subjected to monosaccharide composition analysis and subsequently used for methylation analysis.

### Circular dichroism (CD) spectra

Circular dichroism (CD) spectroscopy (J-815, Jasco, Japan) was performed to assess the structural stability of the polysaccharide. To this end, samples were dissolved in distilled water and scanned across the range of 190–700 nm.

### Congo red test

The reaction of polysaccharides with Congo red and NaOH in different solution systems, combined with scanning wavelength analysis, can reveal whether they have a triple helix structure^16^. Thus, 5 mg of the refined polysaccharide was mixed with 2 mL of distilled water in a test tube. After the polysaccharide was completely dissolved, 2.0 mL of 80 μmol/L Congo red reagent and 1.0 mol/mL NaOH were added to obtain different NaOH concentrations (0, 0.1, 0.2, 0.3, 0.4, and 0.5 mol/mL). After mixing, the solution was left to stand for 5 min, and ultraviolet spectrum analysis in the range of 200–800 nm was performed. The wavelengths of maximum absorbance (*λ*_max_) were collected for analysis. Another set of blank tests was performed using distilled water instead of the polysaccharide solution^34^.

### AFM and scanning electron microscopy (SEM)

AFM analysis (Dimension ICON, Bruker, Switzerland) was conducted by depositing SHP-A on a fresh mica substrate, followed by complete drying. SEM (SU8220, Hitachi, Japan) was subsequently employed to analyze surface morphology^35^.

### Cell viability assays in RAW264.7 mouse macrophages

First, 10 mg of purified SHP-A was accurately weighed to prepare a 1.0 mg/mL stock solution in cell culture medium (DMEM). After filter sterilization, the solution was diluted using DMEM to obtain a gradient of SHP-A concentrations, as follows: 1000 μg/mL (SHP-A), 500 μg/mL (SHP-A1), 250 μg/mL (SHP-A2), 125 μg/mL (SHP-A3), and 62.5 μg/mL (SHP-A4). A 1 × 10^5^/mL cell suspension of cultured macrophages was prepared, and 100 μL of this suspension was added to each well of a 96-well plate. The cells were placed in an incubator and cultured at 37 °C under 5% CO_2_ for 4 h until they adhered to the walls of the well. The blank group (DMEM), control group (DMEM + cells), and treatment groups were established, with each well treated with an equal volume of SHP-A-containing medium. After 24 h of culture, 10 μL of CCK-8 was added to each well, and after 30 min, the absorbance was measured at 450 nm using a microplate reader (iMark, BIO-RAD, USA). Cell viability is calculated according to the following Eq. (3):

3 $$\mathrm{Cell\; viability}\,( \% )=(\mathrm{experimental\; group}-\mathrm{blank\; group})/(\mathrm{control\; group}-\mathrm{blank\; group})\times 100 \%$$

### NO release test in RAW264.7 mouse macrophages

Cells were divided into a control group, positive control group (LPS + macrophages), and treatment groups (macrophages + polysaccharide solutions of different concentrations [1000 μg/mL SHP-A, 500 μg/mL SHP-A1, 250 μg/mL SHP-A2, 125 μg/mL SHP-A3, and 62.5 μg/mL SHP-A4]. A 1 × 10^5^/mL cell suspension of cultured macrophages was prepared, and 1 mL of this suspension was added to each well of a 24-well plate, with three replicates per group. After 24 h of culture with the respective treatment agents, the contents of each well were centrifuged (4 °C, 1000 × *g*), and the supernatant was transferred to a 96-well plate. The content of NO was calculated using a NO assay kit based on the manufacturer’s instructions.

### Neutral red phagocytosis assay in RAW264.7 mouse macrophages

Cells were divided into various groups as described as Section 2.13, and the preparatory steps detailed in Section 2.11 were adopted. Following 24-h of pre-culture in 96-well plates, cellular supernatants were removed prior to polysaccharide treatment. After another 24 h of incubation, the culture medium was replaced with 0.1% (w/v) neutral red solution for 30 min. Subsequently, lysis (Absolute ethanol: acetic acid = 1:1) treatment was performed (100 μL/well, 1 h), followed by spectrophotometric quantification at 450 nm. Phagocytosis rate is calculated according to the following Eq. (4):

4 $$\mathrm{Phagocytosis\; rate\; of\; cells}\,( \% )=\mathrm{experimental\; group}/\mathrm{control\; group}\times 100 \% (\mathrm{Absorbance})$$

### Ovalbumin (OVA) phagocytosis assay in RAW264.7 mouse macrophages

OVA, SHP, and FITC were dissolved in DMSO for 13 h and dialyzed in PBS for 3 days at 4 °C. The dialysate was freeze-dried and named FITC-OVA-SHP-A^36^. After the cell density was adjusted to 1 × 10^5^, the cells were added to 24-well plates containing round coverslips and cultured until they adhered to the wall of the wall. Subsequently, FITC-OVA and FITC-OVA-SHP-A (250 μg/mL) were added to the wells for 12 h, with the OVA content in all samples being the same (250 μg/mL). Then, the culture medium was removed, and the cells were washed twice with PBS before fixing with paraformaldehyde (4%). The paraformaldehyde was washed with PBS, and DAPI was added for 10 min before two more washes with PBS. This procedure was repeated for DID staining, and finally, the cells were observed using confocal laser scanning microscopy (TSC SP8, ICA, Germany).

### Transcriptome sequencing

After verifying RNA purity and integrity, the transcriptome library was prepared with the VAHTS Universal V5 RNA-Seq Library Prep Kit following the manufacturer’s instructions. The library was sequenced on an Illumina NovaSeq 6000 platform to generate 150-bp paired-end reads. Raw data (FASTQ format) were processed with Fastp to remove low-quality reads, and the clean data were retained for subsequent analysis^37^. Subsequently, the hypergeometric distribution algorithm, Gene Ontology (GO), Kyoto Encyclopedia of Genes and Genomes pathway, and other enrichment analyses were performed to analyze DEGs^38^. R (V 3.2.0) and GSEA software were employed to analyze the significant enrichment function items as well as gene enrichment^39^.

### Flow cytometry

The expression of macrophage surface co-stimulators (CD80^+^/CD86^+^) was analyzed using flow cytometry. Macrophages were co-cultured with SHP-A for 12 h, harvested, and stained with anti-mouse CD80^+^/CD86^+^ antibodies prior to detection.

### Quantitative real-time PCR (RT-qPCR)

The gene expression of key transcriptome-identified targets was quantified via RT-qPCR. Total RNA was reverse-transcribed into cDNA using a commercial kit and then amplified in a 25 μL reaction system containing 2 μL cDNA, 12.5 μL TB Green Premix Ex Taq II, 0.5 μL ROX Reference Dye, 8 μL ddH_2_O, and 2 μL primer pairs (Table S1).

### Data and analysis

All experiments were conducted using three biological replicates, and the data were represented as the mean ± standard error of the mean. The data were processed with GraphPad Prism 7.0 using the t-test. The significance criteria were as follows: */# *P* < 0.05, **/## *P* < 0.01, ***/### *P* < 0.001, and ****/#### *P* < 0.0001 (*for comparison with the blank group; #for comparison with the LPS group).

## Supplementary information

Supplementary Material

## Data Availability

The data presented in this study are available on request from the corresponding author.
