# Supplementary material for: Dual-backbone pectic polysaccharide activates NOD1/RIPK2/NF-κB and mitochondrial metabolism to enhance the immune activity of macrophages
Source: NPJ Sci Food. 2025 Oct 8;9:204. doi: 10.1038/s41538-025-00570-0 (PMC12508091; doi:10.1038/s41538-025-00570-0)
Supplement: Supplementary file 1 — Supplementary Material [file 41538_2025_570_MOESM1_ESM.docx]

**Supplementary material**

Dual-backbone pectic polysaccharide activates NOD1/RIPK2/NF-κB and mitochondrial metabolism to enhance the immune activity of macrophages

Ziwei Liu ^1,2^, Hangyu Li ^1,2^, Shuyao Yang^1,2^, Xinnan Zhang ^1,2^, Qianqian Liu ^1,2^, Yuan Xu^1,2^, Yanwen Yang^1,2^, Yi Liao^1,2^, Yao Wang^1,2^, Haibo Feng ^1,2,^*

^1. College of Animal Husbandry and Veterinary Medicine, Southwest Minzu University, Chengdu, Sichuan, 610041, PR China;^

^2. Institute of Qinghai-Tibetan Plateau, Southwest Minzu University, Chengdu, Sichuan, 610041, PR China;^

^* Corresponding author at: College of Animal Husbandry and Veterinary Medicine, Southwest Minzu University, Chengdu, Sichuan 610041, PR China. E-mail address: fenghaiborc@126.com (H. Feng).^

**Table S1.** Mouse primer sequences used for qPCR.

| Gene name | Forward Primer (5´→3´) | Reverse Primer(5´→3´) |
| --- | --- | --- |
| IL1β | GTGTCTTTCCCGTGGACCTTC | TCATCTCGGAGCCTGTAGTGC |
| IL6 | CTTGGGACTGATGCTGGTGAC | TCTCATTTCCACGATTTCCCAG |
| GAPDH | GGGTCCCAGCTTAGGTTCATC | TACGGCCAAATCCGTTCACA |
| NOD1 | GCCCTGAAGCAGAACACCAC | TGCCGTAGCGTCTGGTTCA |
| RIPK2 | CTCCTCGTGTTCCTTGGC | TCCTTGTAGGTTTGGTGC |
| NFKB1 | GAAATCCAACGCAGGGGTCA | AGGCGATGGGTTCCGTCTT |
